# Combination of Polydopamine Coating and Plasma Pretreatment to Improve Bond Ability Between PEEK and Primary Teeth

**DOI:** 10.3389/fbioe.2020.630094

**Published:** 2021-01-29

**Authors:** Rui Teng, Yuchen Meng, Xiaodan Zhao, Jie Liu, Rui Ding, Yilong Cheng, Yunhe Zhang, Yanfeng Zhang, Dandan Pei, Ang Li

**Affiliations:** ^1^Key Laboratory of Shaanxi Province for Craniofacial Precision Medicine Research, College of Stomatology, Xi'an Jiaotong University, Xi'an, China; ^2^School of Chemistry, Xi'an Jiaotong University, Xi'an, China; ^3^Engineering Research Center of Super Engineering Plastics, Ministry of Education, College of Chemistry, Jilin University, Changchun, China; ^4^Department of Periodontology, College of Stomatology, Xi'an Jiaotong University, Xi'an, China

**Keywords:** polyetheretherketone, polydopamine, plasma, bonding properties, biocompatibility

## Abstract

Preformed crowns are preferred to reduce the failure risk of restoration of primary teeth, but some drawback of conventional material is still a main barrier for their clinical use. Polyether etherketone (PEEK), a tooth colored, high-performance thermoplastic polymer, has been recognized as a promising alternative to manufacture the restoration of primary teeth. However, the hydrophobic surface and low surface energy of PEEK make it hard to establish a strong and durable adhesion. In this study, we have evaluated a modification method of polydopamine (PDA) coating with plasma pretreatment for the PEEK films by physical and chemical characterization, bonding properties, and biocompatibility. The surface properties of PEEK were well-characterized by scanning electron microscope (SEM) and X-ray photoelectron spectroscopy (XPS). The adhesive strength of the PEEK films was greatly improved without significant reduction of the proliferation rate of human gingival fibroblast cells in MTT and Live/Dead assays. Therefore, PDA coating with plasma pretreatment may give a new solution for effective clinical application of PEEK in primary performed crowns.

## Introduction

Dental caries in the primary teeth is a highly prevalent public health problem (Ludwig et al., 2014; Innes et al., 2015; Smaïl-Faugeron et al., 2018). The untreated primary dental caries develop rapidly and frequently, leading to discomfort, pain, and further infection (Ortiz et al., 2014; Monte-Santo et al., 2018; Schwendicke et al., 2018; Zeng et al., 2018; Vollu et al., 2019). When dissolution of primary molars progresses into cavitation, placing preformed crowns is a preferred restorative treatment to reduce the failure risk of restoration in the long term, particularly with multi-surface cavities in the clinic (Santamaria et al., 2014; Santamaría et al., 2018; Seale and Randall, 2015; Boyd et al., 2018; Korolenkova and Arzumanyan, 2019; Santamar et al., 2020). Despite recommendations for preformed metal crowns, silver-colored appearance is still a main barrier for their clinical use (Santamaria et al., 2014, 2018; Maciel et al., 2017; Lopez-Cazaux et al., 2019). Moreover, possible metal sensitization is also a contraindication. Recently, zirconia preformed crowns have been developed and used for primary molars (Donly et al., 2018; Mathew et al., 2020; Santamar et al., 2020). However, zirconia preformed crowns may require more tooth tissue removal to create sufficient space for crown placing as well as gingival bleeding on account of the rigid and unbending materials (Innes et al., 2015; Aiem et al., 2017). Besides, the cost of zirconia primary molar crowns is high. All of these limit its clinical use in daily practice (Santamar et al., 2020).

Polyether etherketone (PEEK), as a tooth colored, high-performance thermoplastic polymer, has been considered to be a promising alternative to ceramic materials in crown restoration (Tsuka et al., 2019; Attia and Shokry, 2020; Papathanasiou et al., 2020). PEEK features attractive mechanical properties (similar to dentin and enamel), wear and chemical resistance, dimensional stability, high polishing qualities, good aesthetics, and excellent biocompatibility (Najeeb et al., 2016; Skirbutis et al., 2018; Bathala et al., 2019; Caglar et al., 2019; Tsuka et al., 2019; Papathanasiou et al., 2020). Recently, PEEK has also been applied in clinical dentistry for fixed dental prostheses, implants, abutment, temporary prostheses, and removable prosthodontics (Najeeb et al., 2016; Zoidis et al., 2016; Skirbutis et al., 2018; Papathanasiou et al., 2020). Thus, PEEK should be an ideal and reliable material with the potential to manufacture the restoration of primary molar teeth (Najeeb et al., 2016; Klur et al., 2019).

Effective bonding to PEEK is a prerequisite for its use as a preformed crown material (Tsuka et al., 2019). However, its inert hydrophobic surface and low surface energy make it hard to establish a strong and durable adhesion with dental material (Chaijareenont et al., 2018; Skirbutis et al., 2018). In order to address this issue, several studies have employed different surface-modified methods, such as sand-blasting, silica coating, etching the surface with sulfuric acid, piranha solution, or hydrofluoric acid, to increase the roughness of the PEEK surface for micromechanical interlocking (Zhou et al., 2014; Silthampitag et al., 2016; Caglar et al., 2019; Tsuka et al., 2019). The reactive groups generated by modification may enhance the adhesive strength between PEEK and dental materials through chemical or physical interactions (Schwitalla et al., 2017; Bötel et al., 2018). For example, it has been reported that 98% sulfuric acid etching increased shear bond strength (SBS) more greatly [from 1.75 (0.66) to 27.36 (3.95) MPa] than the aforementioned modifying methods (Chaijareenont et al., 2018). However, the application of the aggressive acidic solutions is not clinically feasible due to the extremely hazardous nature. Hence, exploiting a green and efficient way for the surface modification of PEEK to improve the adhesive property is on demand for the clinical applications in performed crowns.

**Scheme 1:**
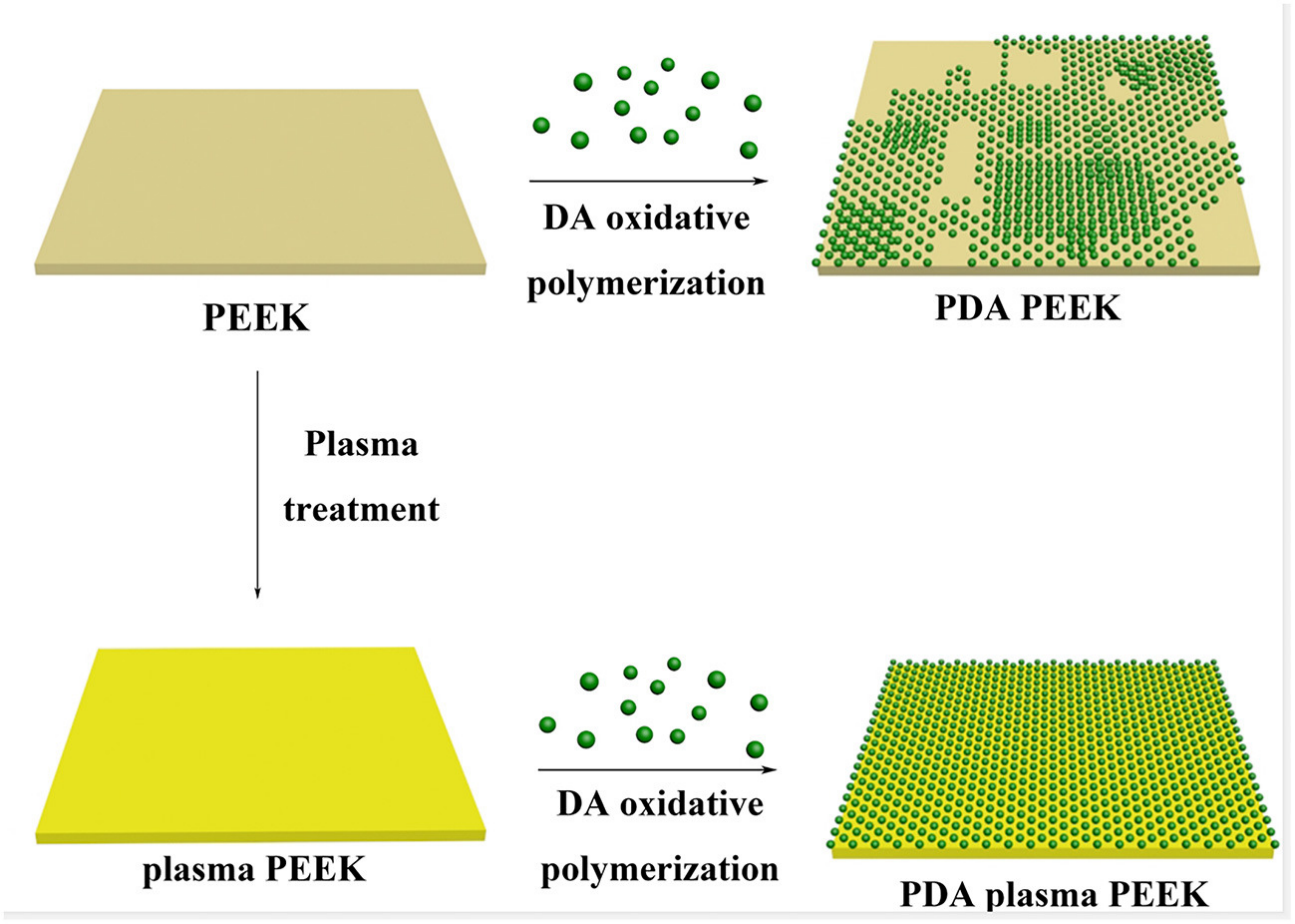
Illustration of the process of PDA deposition on the surfaces of normal PEEK and plasma-treated PEEK.

Dopamine (DA) and its derivatives are attracting materials that have been widely used to functionalize various surfaces for different applications (Lee et al., 2007; Lynge et al., 2011; Liu et al., 2014; Ryu et al., 2018). Immerging materials into the DA solution under alkaline conditions can form a tightly adherent polydopamine (PDA) layer through oxidative polymerization, which can firmly stick to the surfaces of the materials through multiple interaction, including hydrogen bonding, electrostatic interaction, and π-π interaction. In this work, we hypothesize that the deposition of PDA on the surface of PEEK may improve the affinity between PEEK and glass ionomer cement (GIC). In order to further improve the surface modification efficiency, PEEK was processed for plasma treatment, followed by PDA modification. Plasma treatment is a useful technique that can prime any surface for better secondary surface modification, by which carbonyl, hydroxyl, and other groups can be generated to enhance the adhesiveness of surfaces (Zanini et al., 2007; Safinia et al., 2008; Chen and Su, 2011). Through the combination of plasma treatment and PDA deposition (Scheme 1), the adhesive strength between PDA and GIC should be further improved. We carefully studied the surface morphologies of PEEK with different treatments and investigated the adhesive strength of the PEEK films by lap-shear tensile experiments. Furthermore, the SBS of PEEK was also evaluated with dentin of primary molar teeth. MTT and Live/Dead assays were used to test the biocompatibility of the modified PEEK films. Our work may give a new solution to the potential clinical application of PEEK in performed crowns for primary molar teeth.

## Materials and Methods

### Materials

The PEEK films were purchased from Changchun Jilin University Super Engineering Plastics Research Co., Ltd., and the model was 021Film-15. 3-Hydroxytyramine hydrochloride and Tris–HCl were obtained from damas-beta. GIC (Ketac™ CemEasymix) was bought from 3M ESPE (St. Paul, MN, USA). Cell culture materials and chemicals related to evaluating cell viability were purchased from Sigma-Aldrich (St. Louis, MO, USA). The other chemical agents were obtained from Aladdin without further purification.

### Preparation of the PEEK Films

The PEEK films were cut into 5 × 5 cm pieces and washed in an ultrasonic bath for 20 min in ethanol and then in ultrapure water. After drying at 60°C, the PEEK films were kept for further use. The plasma-treated PEEK films were prepared by a plasma surface treatment machine (ZLD-2; Hangzhou Shangqiang Intelligent Technology Co., Ltd., China) for 30 s, which was operated at a frequency of 3 × 10^4^ Hz and a maximum power of about 2 kW. 3-Hydroxytyramine hydrochloride (2 mg/ml) was dissolved into 10 mM Tris–HCl buffer at pH = 8.5. The PEEK films with or without plasma treatment (30 s) were subsequently immersed in the solution for 2, 6, 12, 24, and 48 h, respectively. Continuous stirring was adopted to avoid non-specific deposition. During the process, the color of the solution turned from yellow to dark brown owing to the pH-induced oxidation. After the coating process, the films were washed by ultrapure water to remove the un-conglutinated PDA and dried in the air. The uncoating particles on the surface were wiped off gently.

### Physical and Chemical Characterization

#### Microstructures Observation

Before scanning electron microscope (SEM) analysis, the surfaces of neat PEEK and plasma-treated PEEK with 0, 2, 6, 12, 24, and 48 h PDA coating were mounted on metal stubs using a double-sided conductive tape and vacuum-coated with the gold sputtering layer. The surface morphologies were visually characterized by SEM (Gemini SEM 500; Carl Zeiss, Germany) at ×500 magnification under 20 kV accelerating voltage. All measurements were completed by the same investigator.

#### Elemental Analysis

X-ray photoelectron spectroscopy (XPS) studies were carried out by an AXIS ULTRA photoelectron spectrometer (Kratos Analytical Ltd., Manchester, UK) on the surface of the neat PEEK, plasma-treated PEEK, PDA-coated PEEK (24 h), plasma-pretreated PEEK, and PDA-coated PEEK (24 h). The X-ray source of monochromatized Al Kα (hν = 1,486.7 eV) was operated at 50 W and 15 kV. Elemental compositions (C, O, and N) were determined on the PEEK surfaces. The component peak in the C 1 s spectrum was used as a reference with a binding energy of 284.8 eV. Data analysis was performed using the Kratos spectra deconvolution software (version 2.2.9; Kratos Analytical Ltd., Manchester, UK).

### Bonding Properties

#### Bond Strength Between PEEK Films and Dental Adhesive

Bond strength of PEEK with dental adhesive was tested using lap-shear test. The neat PEEK films and plasma-treated PEEK films with 0, 2, 6, 12, and 24 h PDA coating were cut into rectangular sheets with a dimension of 30 × 10 mm. GIC was adopted as a conventional dental adhesive. The cement agent was mixed strictly following the manufacturer's instructions and evenly applied on the surface of the PEEK films with a bonding area of 10 × 10 mm by the same operator. The test was carried out using a CMT1503 tensile testing machine (Zhuhai SUST Electrical Equipment Co., Ltd, China) at a constant loading rate of 1 mm/min at ambient temperature. Five replicate tests were conducted in each case, and the bond strength was expressed in kPa. The calculation formula is derived by dividing the loading force (N) at the time of fracture by the bonding area (mm^2^).

#### Bond Strength Between PEEK Films and Dentin

Sound primary molar teeth with complete crowns were extracted after informed consent of the donors and their parents from the Department of Pediatric Dentistry, College & Hospital of Stomatology, Xi'an Jiaotong University. The teeth were stored in 0.02% NaN_3_ at 4°C for no longer than 3 months. Mid-coronal dentin disks of 1 mm thick were performed by parallel cuts using a low speed water-cooled diamond saw (Isomet; Buehler, Evanston, IL, USA). The surface of dentin disks was polished with wet 600-grit silicon carbide papers for 1 min to create a standard smear layer. The dimension of each disk was measured with a digital caliper to calculate the bonding area.

The adhesive procedure was performed with the GIC following the instructions and applied on the dentin surface evenly. The neat PEEK films, plasma-treated PEEK films, PDA-coated PEEK films (24 h), plasma-pretreated PEEK films, and PDA-coated PEEK films (24 h) were placed carefully to ensure complete contact with each dentin disk. The SBS tests were performed on a universal test machine at a speed of 1 mm/min. The bonding strength value (kPa) was determined by dividing the loading force (N) at the time of fracture by the bonding area (mm^2^). Five replicate tests were conducted for each group.

### *In vitro* Biocompatibility

#### Cell Culture

Primary human gingival fibroblast cells (HGF-1; ATCC, VA, USA) were used to assay the biocompatibility of the PEEK films. Cells were cultured in dishes with growth medium at 37°C in air with 95% humidity plus 5% CO_2_, and the medium was changed every 2–3 days. The growth medium consisted of 89% α-minimum essential medium, 10% fetal bovine serum, and 1% antibiotic (100 U/ml streptomycin and 100 U/ml penicillin). The cells were passaged after reaching 80–90% of confluence. The passages 3–5 of the cells were used in the present study.

#### Preparation of Extracts From the PEEK Films

In order to simulate the physiological environment of the oral cavity, the extracts of the PEEK films were adopted to demonstrate biocompatibility. All the samples were prepared according to the ISO-10993-12:2012. The neat PEEK films, plasma-treated PEEK films, PDA-coated PEEK films (24 h), plasma-pretreated PEEK films, and PDA-coated PEEK films (24 h) were cut into square sheets with a dimension of 10 × 10 mm under completely aseptic conditions and then exposed to UV light for 1 h for sterilization. For each group, every three samples were transferred to a sterile cell-culture 24-well plate, and 1 ml of the growth medium was added to each well, and then were cultured at 37°C in air with 95% humidity plus 5% CO_2_. Finally, the extracts were collected at 3 days.

#### MTT Assay

The cells were seeded into sterile 96-well plates at 2 × 10^3^ per well in the growth medium as described above and followed to attach for 24 h at 37°C under 5% CO_2_. After treated with a series of the extracts for 24, 48, or 72 h, the cells were added at a final concentration of 0.5 mg/ml MTT and incubated for 4 h. After removing the medium, 150 μl of DMSO was added. The absorbance was determined at 490 nm, and the cell viabilities were expressed as a percentage of the control. All samples were performed in triplicate.

#### Live/Dead Staining

The viability of the cells was evaluated using a Live/Dead kit according to the manufacturer's protocol. The cells were planted into 24-well plate with a density of 2 × 10^4^ per well and cultured in the growth medium for 24 h. After replacing the growth medium with fresh extract from the PEEK films, the cells were further cultured for 24 h. Then, the cells were stained with 500 μl of calcein-AM/propidium iodide dye for 15 min and observed under a fluorescent microscope (DMi8; Leica, Germany) for the green (492 nm) and red (545 nm) fluorescence. All experiments were performed in triplicate.

### Statistical Analysis

SPSS software (v18; IBM, Chicago, IL, USA) was used to analyze data. Data of bonding properties analysis were presented as mean ± standard deviation. For comparing the bond strength of neat PEEK and plasma-pretreated PEEK with 0, 2, 6, 12, and 24 h PDA coating, two-way analysis of variance (ANOVA) followed by Tukey's *post-hoc* test was conducted to evaluate the significant differences among groups. Statistically significant differences (*P*) of cell viabilities among groups were measured using one-way ANOVA followed by Tukey's multiple-comparison analysis. The level of significant differences was set in advance at *P* < 0.05.

## Results and Discussion

**Figure 1:**
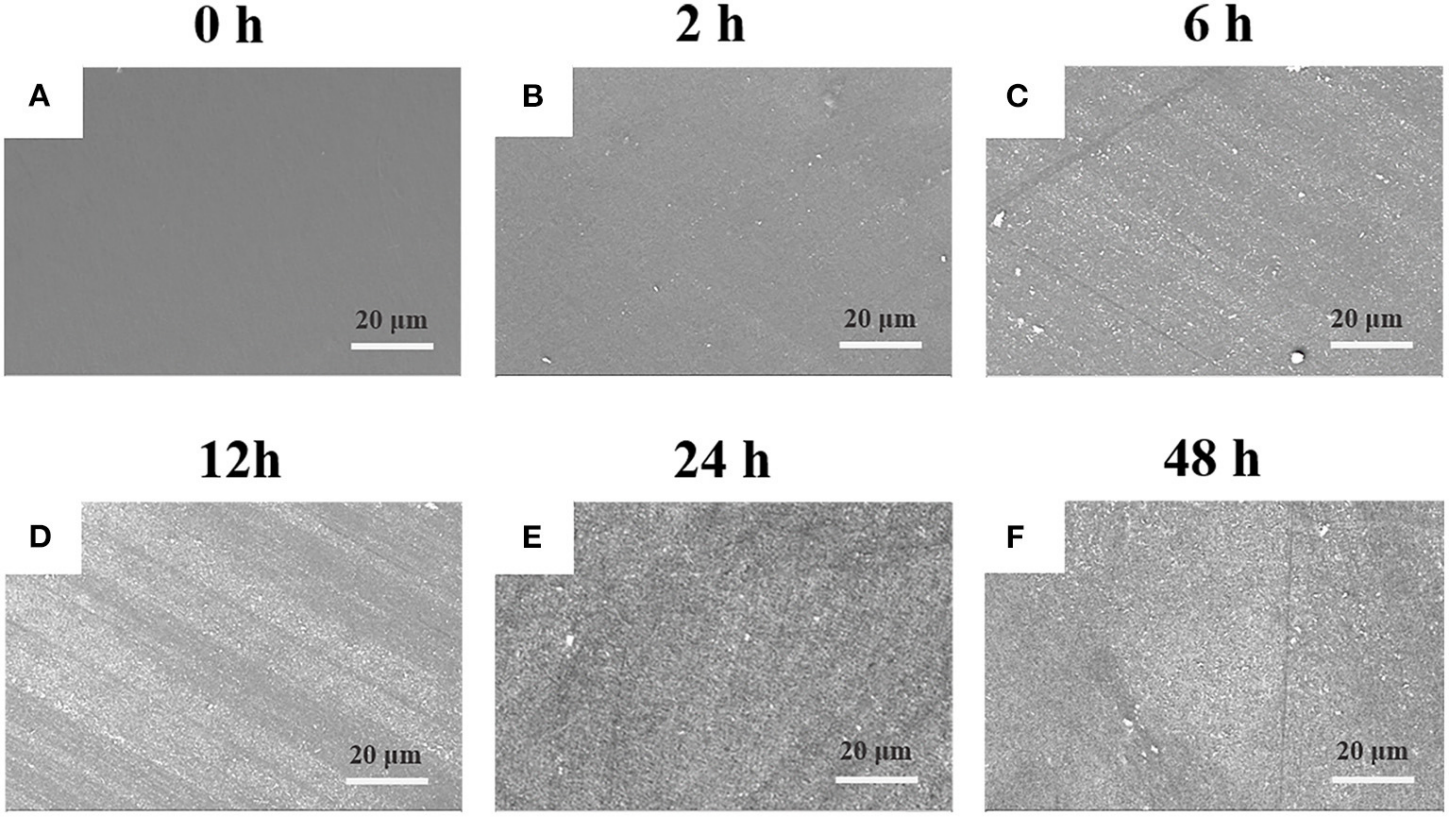
SEM images of plasma-treated PEEK coating with PDA film for **(A)** 0, **(B)** 2, **(C)** 6, **(D)** 12, **(E)** 24, and **(F)** 48 h.

The PEEK film was immersed into DA solution at room temperature under basic condition to initiate the self-oxidative polymerization for surface modification (Lee et al., 2007). As shown in Supplementary Figure 1, it was found that there was a small amount of PDA particles deposited on the surface of PEEK with the treatment for 2 h compared with the neat PEEK film, and that the surface tended to be rough. With the increase of incubation time, the deposited PDA particles gradually increased, and there was no obvious difference observed between 24 and 48 h (Supplementary Figure 1), which was consistent with the previous work for the modification of polyvinylidene fluoride (PVDF) film (Jiang et al., 2011). Moreover, we found that the PDA particles aggregated into large clusters for 24 h incubation, which made the PEEK film surface rougher and more non-uniform and may be beneficial for the improvement of the adhesive behaviors of PEEK. Comparatively, with the prior treatment by plasma, the deposition of PDA on the surface was significantly enhanced (Figure 1). It is worthy to note that there was no difference on the surface morphology observed after plasma treatment compared with the neat PEEK film. Since there were polar groups generated after plasma treatment, the non-covalent interactions between PDA and modified PEEK, such as electrostatic interaction and hydrogen bonding, should be enhanced, which may lead to more PDA deposition (Thakur et al., 2014). We found that a uniform deposition of PDA particles was observed, and that the PEEK surface was almost fully covered after incubation for 24 h, indicating the efficient coating of PDA on the plasma-treated PEEK surface (Figure 1). Representative photographs of neat PEEK, plasma-treated PEEK, PDA-coated PEEK (24 h), plasma-pretreated PEEK, and PDA-coated PEEK (24 h) were listed in Supplementary Figure 2. Macroscopically, there was no difference in appearance for the neat and plasma-treated PEEK films, and both showed uniform light-yellow surfaces. The surface of the PEEK film coated by PDA with/without plasma pretreatment turned from yellow to dark brown as the modifying time increases, and it is related to the self-oxidative polymerization of DA similar to the formation of melanin (Lee et al., 2007).

**Figure 2:**
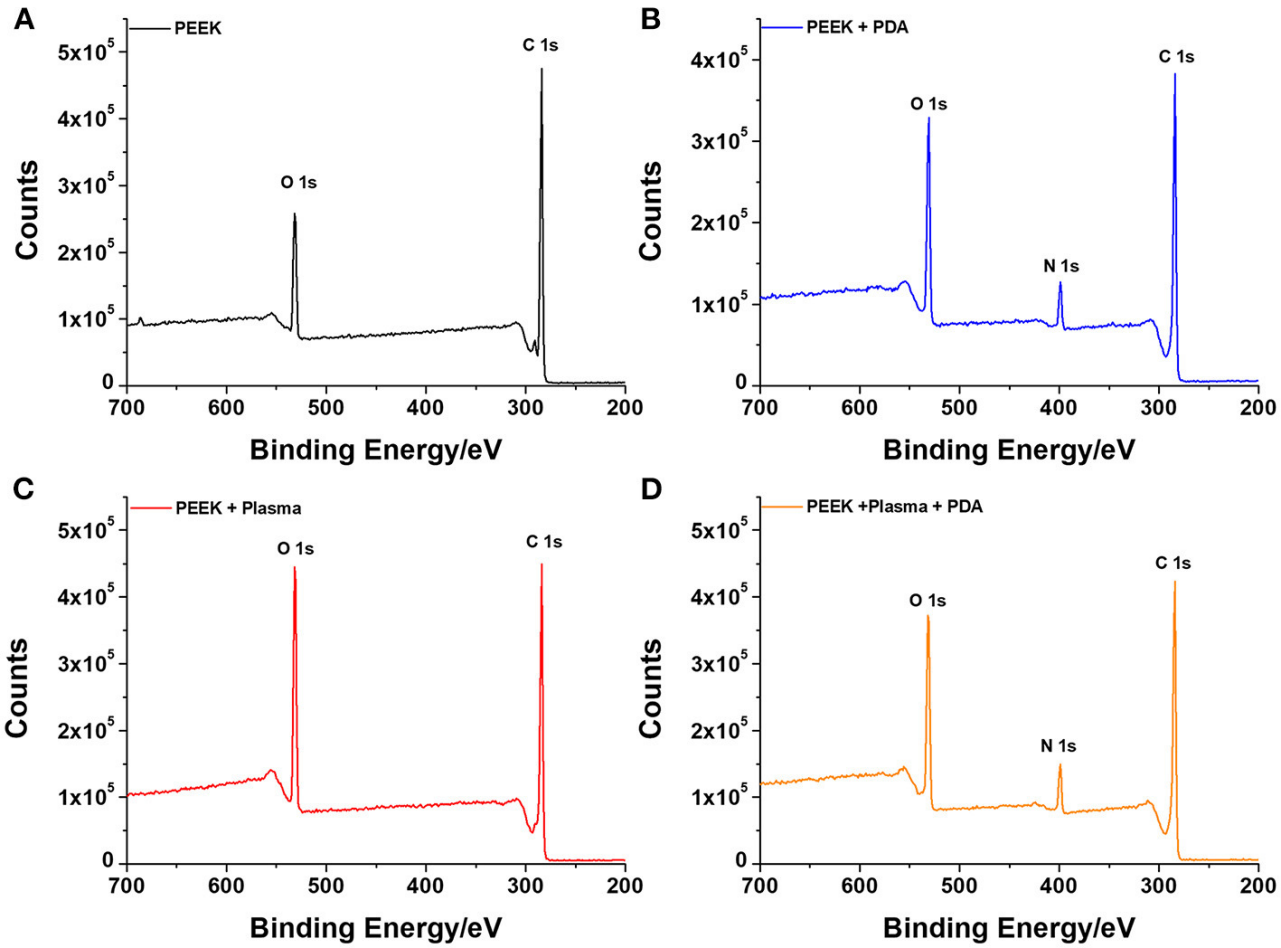
XPS analysis of the various surfaces with or without different treatments for **(A)** neat PEEK, **(B)** PDA-coated PEEK (24 h), **(C)** plasma-treated PEEK, and **(D)** plasma-treated PEEK coating with PDA (24 h).

To further confirm the successful deposition of PDA on the surface of PEEK, XPS analysis was employed to characterize the surface chemical compositions. Figure 2 shows the survey scan spectra of neat PEEK, PDA-coated PEEK, plasma-treated PEEK, and PDA-coated PEEK after plasma treatment. It was found that two separated peaks that correspond to C 1s (285 eV) and O 1s (532 eV) were detected in all the surfaces of the PEEK films, and that there was no new peak observed when the film was treated with plasma. However, after PDA deposition, a distinct peak at 400 eV attributed to N 1s appeared, indicating the successful coating of PDA. This is consistent with the results by SEM observation. Furthermore, the deconvolution of the narrow scan C1s peak shown in Supplementary Figures 3A,B indicated that the saturated hydrocarbon C–C/C–H peak decreased from 73.25 to 61.44% as well as the increase of C–O (from 17.15 to 23.35%) and C=O (from 3.00 to 10.38%) peaks after plasma treatment, suggesting the introduction of oxygen functionalities and the increase in carboxyl groups on the surface. Plasma-assisted modification results in the formation of reactive functional groups on the surface, thus facilitating the effective surface functionalization of PEEK by PDA (Thakur et al., 2014). Hence, there was more PDA deposited on the PEEK film surface owing to the application of plasma.

Based on the surface modification results, we speculate that the resulted rough surface may be beneficial to the improvement of the bond strength between PEEK and primary teeth. GIC is the material of choice for treatment of primary teeth in several countries due to the properties of fluoride release and good biocompatibility, which may reduce secondary caries progression, as well as the simple clinical application. It has been confirmed that there is a stable chemical bond formed between GIC and teeth surfaces (Bonifácio et al., 2012; Alves et al., 2013). Therefore, GIC was applied in the evaluation of the bond strength between PEEK films and primary teeth.

**Figure 3:**
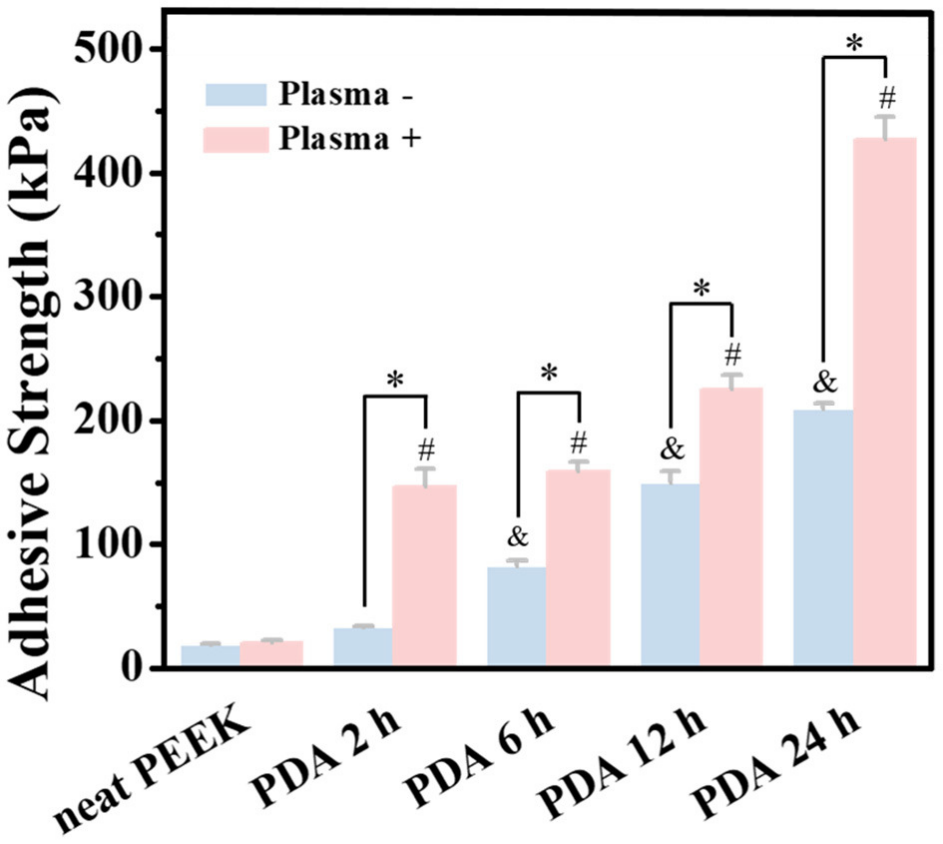
Bonding strength tests of PEEK and plasma-treated PEEK coating with PDA in lap-shear test. *A statistically significant difference was observed at the same PDA-coating time (*P* < 0.05). ^&^A statistically significant difference was observed compared with the neat PEEK (0 h) in the group without plasma pretreatment (*P* < 0.05). ^#^A statistically significant difference was observed compared with the plasma-treated PEEK (0 h) in the plasma-pretreated group (*P* < 0.05).

First, lap-shear experiments were employed to qualify the bond strength of GIC and PEEK films with different treatments. Because of the inertness of the neat PEEK film, poor adhesiveness to dental adhesive (with 18.4 ± 0.9 kPa bond strength) was detected (Figure 3). During the test, the two PEEK films bonded by dental luting cement were very easy to disconnect. In addition, we did not find obvious improvement in the bond strength for the plasma-pretreated PEEK films (20.46 ± 1.7 kPa) and 2 h of PDA coating (32.4 ± 1.8 kPa) compared with the neat PEEK group. However, after PDA treatment for 6, 12, and 24 h, the bond strength was significantly increased to 82.2 ± 4.2, 149.2 ± 9.5, and 209.4 ± 4.4 kPa, respectively. The PDA deposition significantly enhanced the PEEK films' adhesive effect with dental luting cement. Furthermore, the combination of plasma pretreatment and further PDA coating greatly boosted the bond strength. The bond strength corresponding to 2, 6, 12, and 24 h PDA polymerization after plasma treatment was further significantly increased to 146.3 ± 14.5, 158.6 ± 7.9, 225.1 ± 11.5, and 427.0 ± 18.3 kPa, respectively. Surface topography affected the bond strength between the dental luting cement and the PEEK films due to the micromechanical locking force (Silthampitag et al., 2016). The improvement for bond strength may be attributed to more PDA deposition on the PEEK films, leading to the rougher surfaces. It is worthy to mention that the bond strength obtained by the PEEK films with PDA coating for 24 h was 11 times higher than that by the neat PEEK films, which was elevated to 14 times by the intervention of plasma treatments. A significant similar increase in bond strength between PEEK and resin composites was previously observed by 90 or 98% sulfuric acid etching (Chaijareenont et al., 2018). The high concentration of sulfuric acid etching was still considered to be the most recommended treatment to modify the surface of PEEK to improve bonding (Zhou et al., 2014; Silthampitag et al., 2016). The result of the lap-shear experiments demonstrated that the PDA coating with plasma pretreatment may give a good solution to the bonding of PEEK.

**Figure 4:**
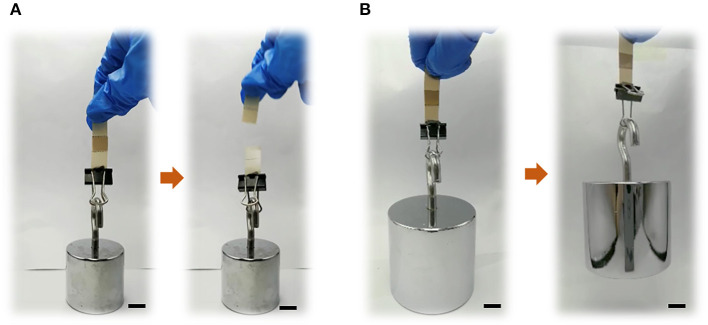
Bond strength exhibitions of **(A)** neat PEEK (loading 200 g) and **(B)** plasma-treated PEEK coating with PDA for 24 h (loading 2 kg). Scale bar = 10 mm.

Furthermore, to verify our hypothesis, the two PEEK films bonded using GIC (adhesive area: 10 × 10 mm) were processed to lift different weights to observe the bonding stability. As shown in Figure 4A and Supplementary Movie 1, it was found that the bonded neat PEEK films were too fragile to withstand the weight of 200 g. Comparatively, the plasma-pretreated and PDA-coated PEEK films can constantly lift 2 kg weight (Figure 4B and Supplementary Movie 2), demonstrating the significant increase of the adhesiveness between dental luting cement and PEEK films.

**Figure 5:**
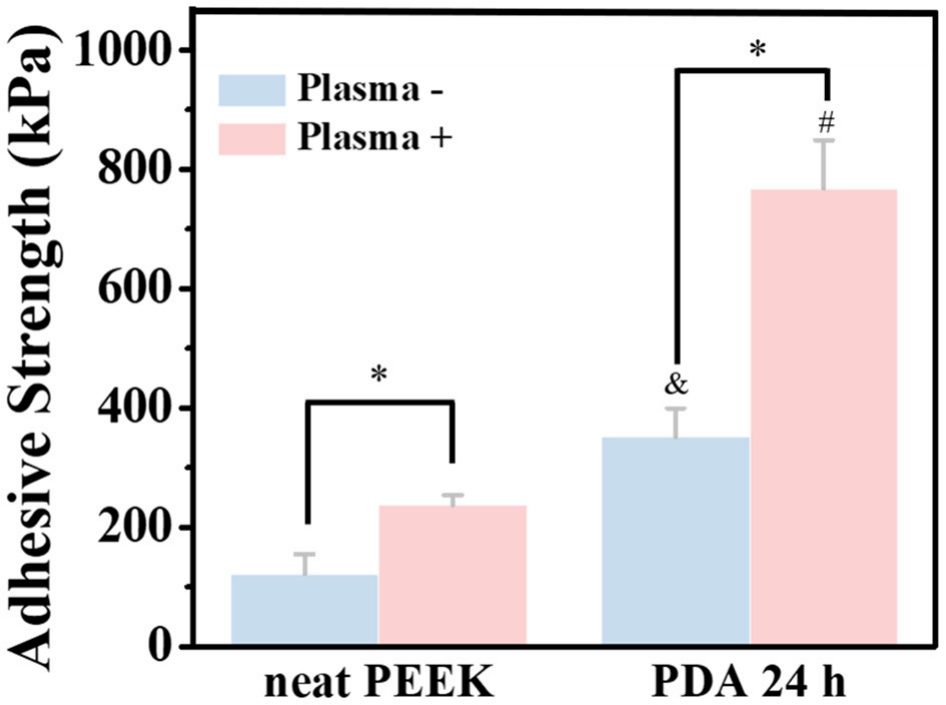
Bonding strength of normal PEEK and plasma-treated PEEK coating with PDA with dentin disk of primary molar teeth. *A statistically significant difference was observed at the same PDA-coating time (*P* < 0.05). ^&^A statistically significant difference was observed compared with the neat PEEK (0 h) in the group without plasma pretreatment (*P* < 0.05). ^#^A statistically significant difference was observed compared with the plasma-treated PEEK (0 h) in the plasma-pretreated group (*P* < 0.05).

To mimic the practical applications, the bond strength of the PEEK films with or without surface modification to dentin disk of primary molar teeth was also tested (Figure 5 and Supplementary Figure 4). The bond strengths of the neat PEEK films with or without plasma treatment to dentin disks were 122.3 ± 32.7 and 237.3 ± 16.9 kPa, respectively. Since there were various polar groups generated on the surface of the PEEK film with plasma treatment, it is predictable that the bond strength can be significantly enhanced by plasma treatment. Moreover, after PDA coating for 24 h on the PEEK film, the bond strength was significantly increased to 351.9 ± 47.5 kPa. As expected, the PEEK film with both plasma treatment and PDA coating for 24 h exhibited the strongest bonding (767.8 ± 81.5 kPa). These results suggested that our methods for the PEEK film modification may be useful for the clinical applications. Moreover, a previous result confirmed that the PDA-coating process was performed after plasma pretreatment for the reinforcement of the adhesive force and the stability of the PDA-coating layer on the polytetrafluoroethylene (PTFE) substrates (Cheng et al., 2019). Therefore, the modification of PDA coating with plasma pretreatment on the PEEK surface may have great potential to provide an effective and durable bond with primary molar teeth.

**Figure 6:**
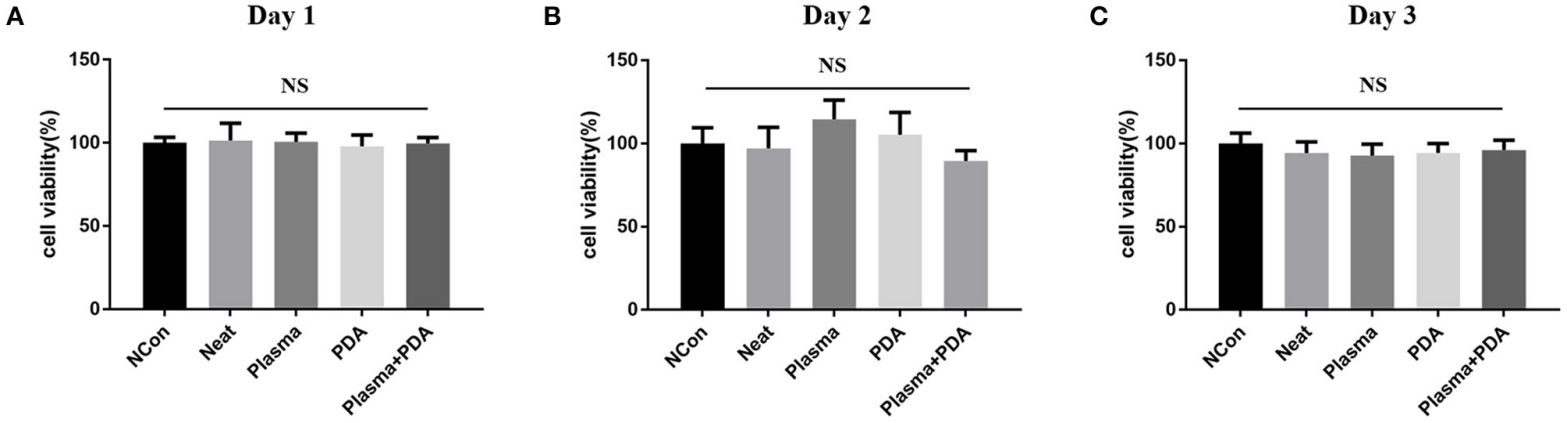
Cell proliferation was determined using MTT assay for **(A)** 24, **(B)** 48, or **(C)** 72 h. NS (*P* > 0.05).

**Figure 7:**
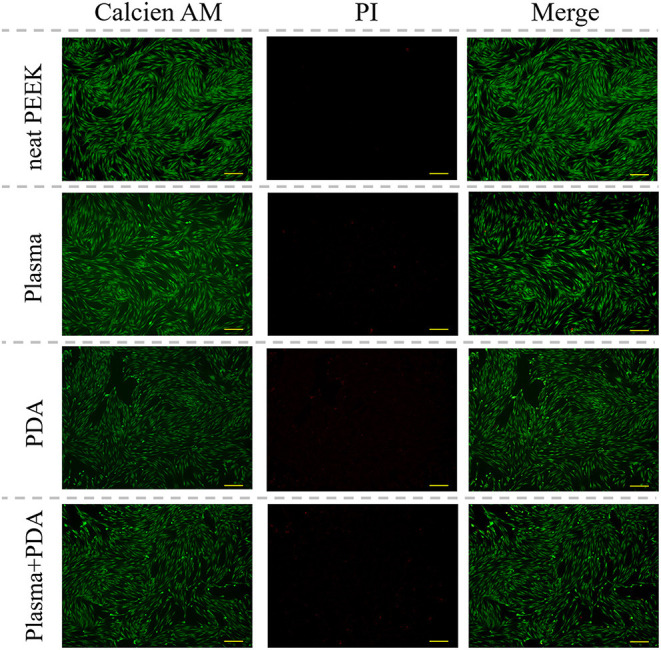
Representative microscopic fluorescence images of live/dead staining specimens for 24 h. Scale bar = 163 μm.

The cell biocompatibility of the PEEK films was evaluated by MTT assay and live/dead staining. As shown in Figure 6, there was no obvious difference found among the neat PEEK group, PDA-coated PEEK (24 h), plasma-treated PEEK, and PDA-coated PEEK (24 h) after the plasma treatment groups in the MTT assay at 1, 2, and 3 days. Afterward, the live/dead staining was used to visually observe the cell biocompatibility. The live and dead cells were stained with green and red fluorescence, respectively. It was found that the majority of the cells presented normal morphology in all the PEEK groups (Figure 7). Therefore, these results showed that the modified PEEK films own good cell biocompatibility and should be safe for further *in vivo* application.

## Conclusion

Modified PEEK by PDA coating for different times with/without plasma pretreatment was fabricated and well-characterized using SEM and XPS. The modification treatments had a significant impact on the bonding properties of the PEEK surfaces without causing a significantly decreased proliferation rate of human gingival fibroblast cells. Among those treatments, PDA coating for 24 h with plasma pretreatment seems to be most effective with obvious improvement of adhesive strength between modified films and dentin disk of primary molar teeth. Our work provides a green, safe, and effective way for the modification of the PEEK surface and may give a new solution for the employment of PEEK primary performed crowns in potential clinical applications.

## Data Availability Statement

The original contributions presented in the study are included in the article/Supplementary Material, further inquiries can be directed to the corresponding author/s.

## Author Contributions

DP and AL supervised the study. RT, YM, XZ, JL, and RD performed the experiments and discussed the results. YM and XZ conducted the evaluation of bonding properties and SEM and XPS analyses. JL and RD participated in biocompatibility experiments. RT wrote the first draft of the manuscript. YC, YuZ, YaZ, DP, and AL coordinated the work, designed the experiments, and performed its final revision. All authors contributed to the article and approved the submitted version.

## Conflict of Interest

The authors declare that the research was conducted in the absence of any commercial or financial relationships that could be construed as a potential conflict of interest.
