# Supplementary material for: Combination of Polydopamine Coating and Plasma Pretreatment to Improve Bond Ability Between PEEK and Primary Teeth
Source: Front Bioeng Biotechnol. 2021 Jan 29;8:630094. doi: 10.3389/fbioe.2020.630094 (PMC7880054; doi:10.3389/fbioe.2020.630094)
Supplement: Supplementary file 1 [file Presentation_1.pptx]

## Slide 1
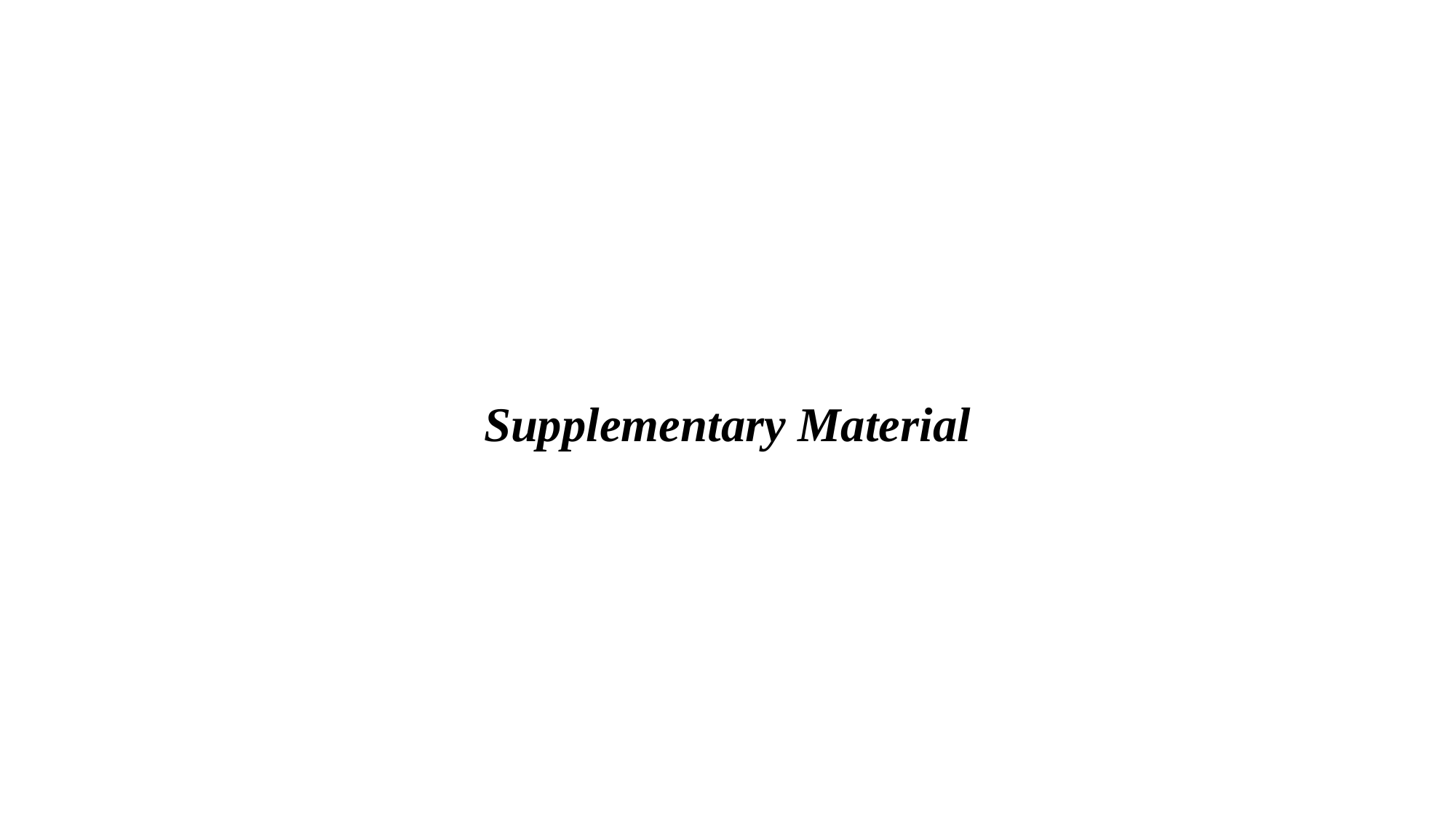

Supplementary Material

## Slide 2
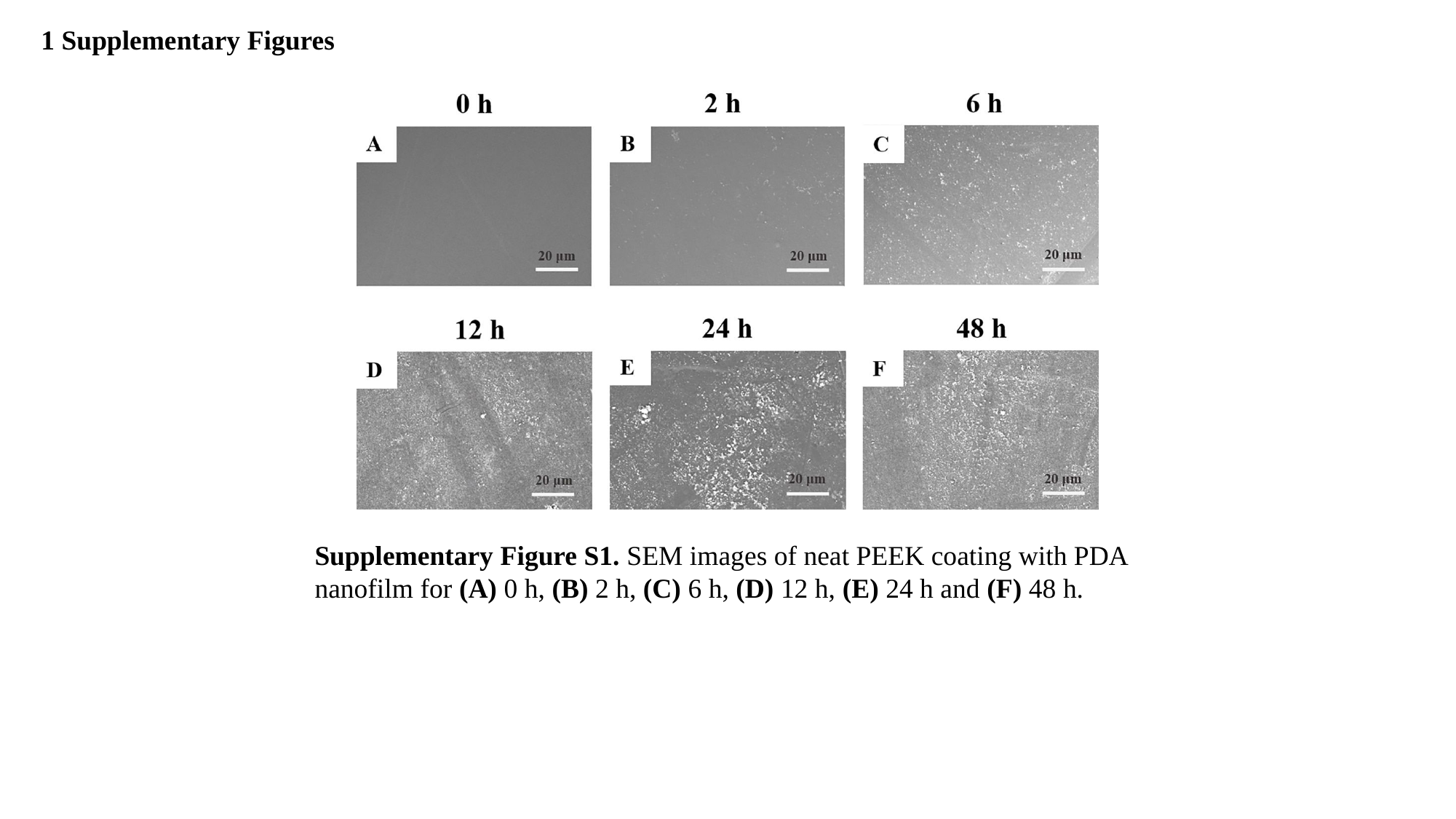

1 Supplementary Figures
Supplementary Figure S1. SEM images of neat PEEK coating with PDA nanofilm for (A) 0 h, (B) 2 h, (C) 6 h, (D) 12 h, (E) 24 h and (F) 48 h.

## Slide 3
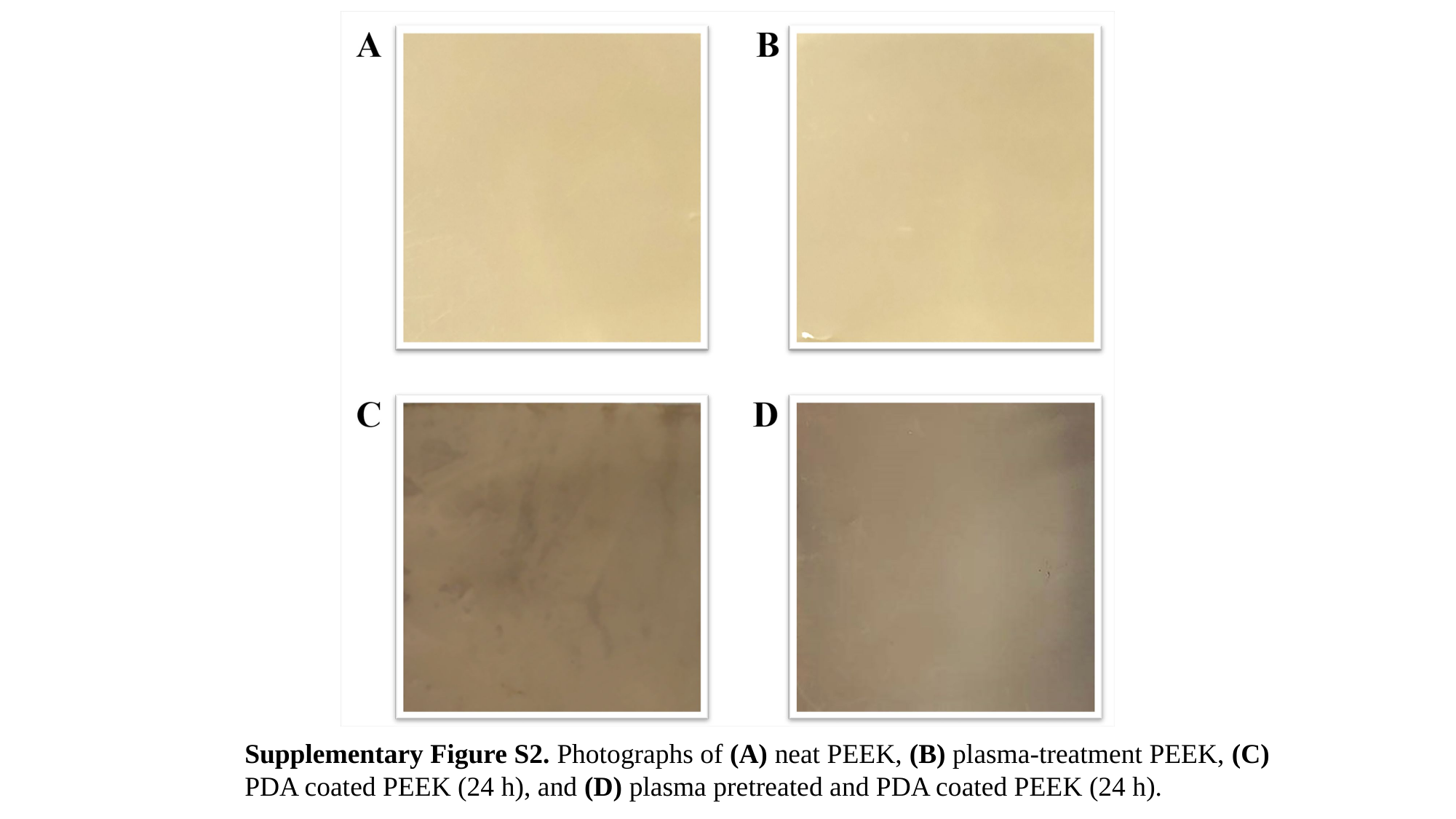

Supplementary Figure S2. Photographs of (A) neat PEEK, (B) plasma-treatment PEEK, (C) PDA coated PEEK (24 h), and (D) plasma pretreated and PDA coated PEEK (24 h).

## Slide 4
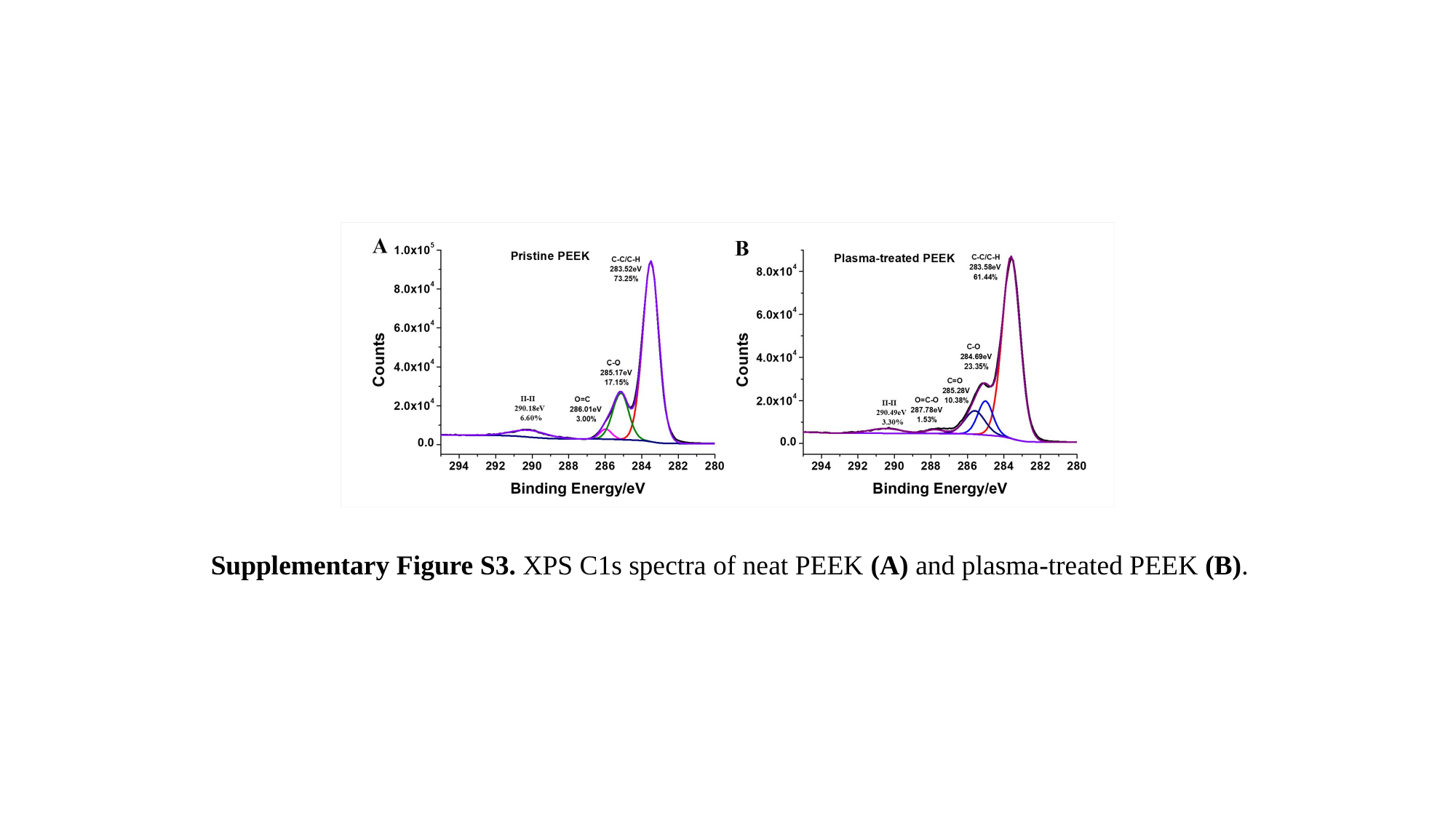

Supplementary Figure S3. XPS C1s spectra of neat PEEK (A) and plasma-treated PEEK (B).

## Slide 5
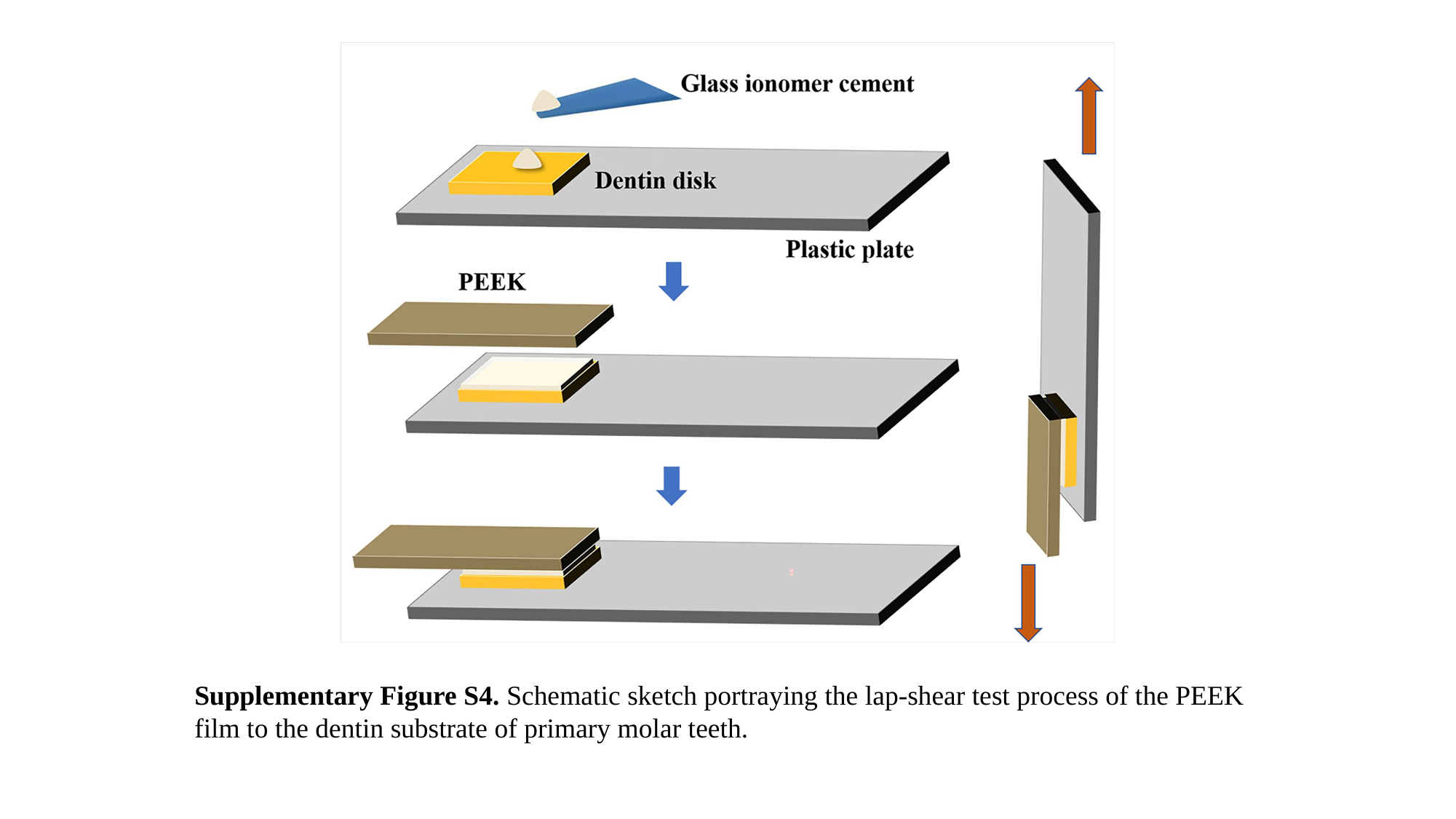

Supplementary Figure S4. Schematic sketch portraying the lap-shear test process of the PEEK film to the dentin substrate of primary molar teeth.

## Slide 6
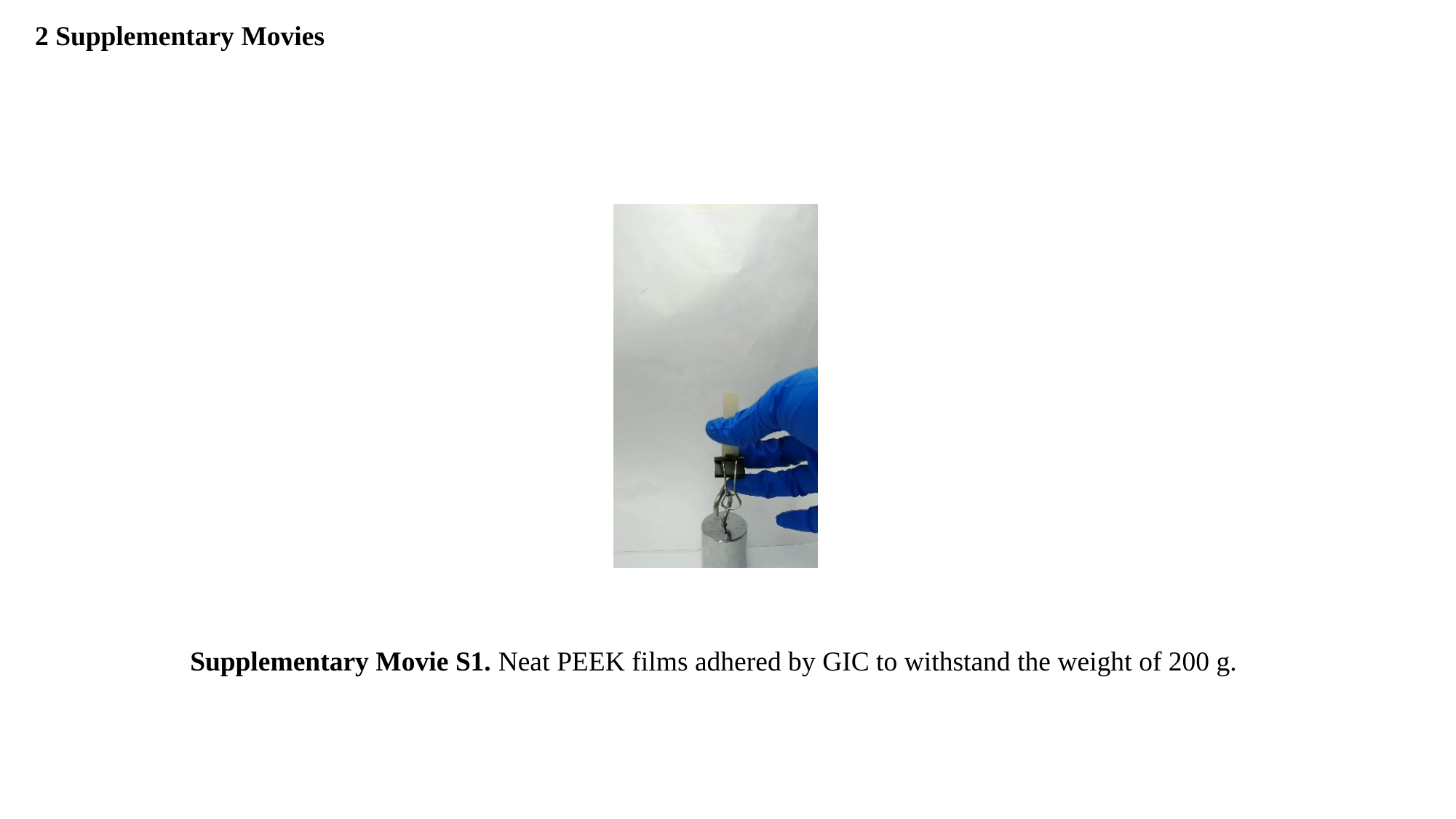

2 Supplementary Movies
Supplementary Movie S1. Neat PEEK films adhered by GIC to withstand the weight of 200 g.

## Slide 7
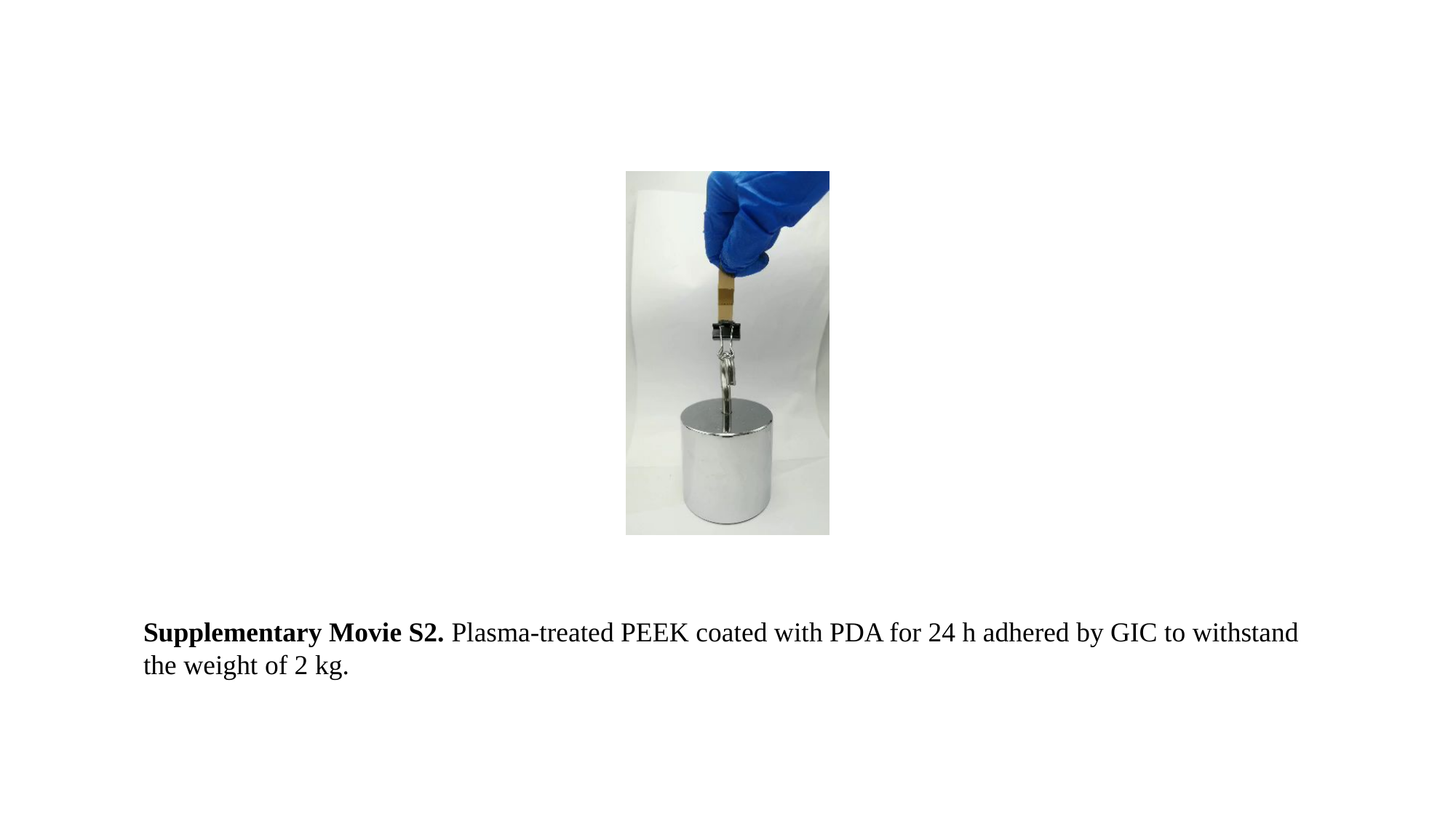

Supplementary Movie S2. Plasma-treated PEEK coated with PDA for 24 h adhered by GIC to withstand the weight of 2 kg.
